# Aircraft Wake Vortex Recognition Method Based on Improved Inception-VGG16 Hybrid Network

**DOI:** 10.3390/s25092909

**Published:** 2025-05-04

**Authors:** Weijun Pan, Yuhao Wang, Leilei Deng, Yanqiang Jiang, Yuanfei Leng

**Affiliations:** 1 School of Air Traffic Management, Civil Aviation Flight University of China, Guanghan 618307, China; wangyuhao496@gmail.com (Y.W.); deng_leilei@cafuc.edu.cn (L.D.); 2 School of Transportation and Logistics, Southwest Jiaotong University, Chengdu 611756, China; tower_c_j@163.com; 3 School of Electronic and Information Engineering, Beihang University, Beijing 100191, China; ffxdds@163.com

**Keywords:** wake vortex, wake vortex identification, inception-VGG16, Doppler lidar, deep learning

## Abstract

This paper proposes a hybrid deep learning network architecture (Inception-VGG16) to address the challenge of accurate aircraft wake vortex identification. The model first employs a Feature0 module for preliminary feature extraction of two-dimensional Doppler radar radial velocity data. This module comprises convolution, batch normalization, ReLU activation, and max pooling operations. Subsequently, improved InceptionB and InceptionC modules are utilized for parallel extraction of multi-scale features. The InceptionB former module adopts two parallel branches, combining 1 × 1 and 3 × 3 convolutions, and outputting 64-channel feature maps, while the InceptionC latter module expands the number of channels number to 128, enhancing the model’s feature representation capability. The backend employs the VGG16’s hierarchical structure, performing deep feature extraction through multiple convolution and pooling operations, and ultimately achieving wake vortex classification through fully connected layers. Experimental validation based on 3530 wind field samples collected at the Chengdu Shuangliu Airport demonstrates that compared to traditional methods (SVM, KNN, RF) and single deep networks (VGG16), the proposed hybrid model achieves a classification accuracy of 98.8%, significantly outperforming comparative traditional methods (SVM, KNN, RF) and single deep networks (VGG16). The model not only overcomes the limitations of single networks in processing multi-scale wake features but also enhances the model’s ability to identify wake vortices in complex backgrounds through deep feature hierarchies, providing a new technical solution for aviation safety monitoring systems based on deep learning.

## 1. Introduction

Aircraft wake vortices are an inevitable phenomenon that accompany the generation of lift during aircraft operations. The pressure difference between the upper and lower surfaces of the wings of an aircraft flying at a high speed creates intense rotational airflow at the wingtips. This vortex structure extends backward and downward for several kilometers, and is characterized by long duration and a large impact range [1]. There are serious risks associated with wake vortices during low-altitude flight phases. When a following aircraft inadvertently enters the wake vortex region of a leading aircraft, the strong rotational forces on the trailing aircraft’s wings may cause severe rolling moments, leading to attitude instability or uncontrolled descent. Such scenarios pose a significant threat to aviation safety, affecting airport operational efficiency and, in extreme cases, can potentially cause catastrophic accidents [2]. Therefore, it is of great significance to accurately identify and provide timely warnings for wake vortices.

The rapid growth of the global aviation industry has made it critical to accurately identify and monitor aircraft wake vortices in real-time for ensuring aviation safety. Breitsamter [1] conducted a comprehensive review of the physical characteristics and impacts of aircraft wake vortices. The author examined the evolution mechanisms from near-field to far-field vortices and explored the mitigation strategies, thereby providing a theoretical foundation for this field. Gharbia et al. [2] summarized the development of wingtip vortex mitigation techniques over the past 60 years. Panagiotou et al. [3] used smoke flow visualization and 3D laser Doppler velocimetry to validate the effectiveness of winglets in suppressing vortex intensity.

Among traditional measurement and analysis methods, Köpp et al. [4] pioneered a new direction for wake vortex monitoring by introducing a wake vortex parameter measurement system based on a 2-micron pulsed Doppler lidar in 2004. Holzäpfel et al. [5] systematically evaluated various calculation methods for calculating the circulation of aircraft wake vortices observed by lidar, revealing the systematic errors and applicability of different approaches. Liu et al. [6] proposed a wake vortex localization method that combined radial velocity and spectral width by analyzing the evolution characteristics of wake vortices under different crosswind conditions. Rojas et al. [7] developed the CGM (circulation generator module) tool for wake vortex circulation calculation, providing essential references for the safety assessments of wake vortex encounters. Recently, Michel et al. [8] developed a short-pulse lidar system that could achieve a spatial resolution of 22.5 meters, offering new technical means for precise wake vortex center localization.

Given the rapid advancement of artificial intelligence technologies, Shakirov et al. [9] provided a comprehensive review of the state of deep learning. He, K. et al. [10] proposed ResNet, introducing a residual learning framework to address the deep network training degradation problem. Szegedy et al. [11] achieved a 3.08% top-five error rate in the ImageNet classification task with Inception-v4 and Inception-ResNet. Huang et al. [12] introduced DenseNet, which enabled efficient feature reuse through dense connections. Liu et al. [13] proposed CBNet, which used composite connections to significantly enhance the object detection performance. Zhang et al. [14] theoretically analyzed the advantages of adaptive optimization methods in attention models and introduced the novel ACClip algorithm. These foundational studies have provided significant technical support for wake vortex recognition tasks.

In specific applications relating to wake vortex recognition, Luo et al. [15] studied the evolution characteristics of A333 wingtip vortices using large eddy simulations and dynamic adaptive grid techniques. Pan, W et al. [16] constructed the Wind3D-6000 lidar wake vortex dataset based on an improved VGG16 network, achieving a recognition accuracy of 0.984. Xu et al. [17] proposed MRFF-YOLO, which used multi-receptive field feature fusion to achieve significant performance improvements on remote sensing datasets. Shen et al. [18] innovatively used the YOLOv5s network to introduce the concepts of single vortex localization and intensity-based classification criteria.

In recent studies, Chu et al. [19] developed a two-stage probabilistic deep learning framework that achieved high-precision wake vortex localization and intensity prediction. Ma et al. [20] proposed the YOLOv8n-CBAM-EfficientNetV2 model, which achieved a detection accuracy of 96.59% and real-time performance of 250 frames per second. Wei et al. [21] demonstrated that their inversion model based on the DBN and GA-BPNN outperformed traditional methods in real airport environments. Zhang et al. [22] integrated Doppler lidar data to achieve rapid wake vortex parameter recognition under various weather conditions. Pan, W et al. [23] proposed an improved GoogLeNet model, which achieved a recognition accuracy of 98.44% and a detection speed of 160 fps. However, these existing methods still face several challenges as described below:

1. Single-network architectures often have limited capabilities to handle multi-scale wake vortex features.
2. Feature extraction and noise suppression capabilities require further improvement under complex backgrounds.
3. It is difficult to balance real-time performance and accuracy within a single model.

To address these aforementioned issues, this paper proposes a hybrid deep learning architecture that combines the strengths of the Inception and VGG16 networks. The proposed model leverages improved InceptionB and InceptionC modules to enable parallel extraction of multi-scale features; in parallel, it also utilizes the hierarchical structure of VGG16 to enhance deep feature representation. This innovative design provides a new technical solution to achieve improved wake vortices recognition performance in complex environments.

The paper is structured as follows: Section 2 introduces the wake vortex generation principles and Doppler lidar detection principles, along with a detailed description of the on-site wake detection experiments; Section 3 proposes the wake vortex recognition method based on the improved Inception-VGG16 hybrid network, elaborating on the design and implementation of the Feature0 module, improved InceptionB and InceptionC modules, and the classifier module; Section 4 presents the experimental results based on the Chengdu Shuangliu Airport dataset and a performance comparison with traditional methods; Finally, Section 5 summarizes the research findings and discusses future research directions.

## 2. Materials and Methods

### 2.1. Principle of Wake Generation

The aircraft wake system primarily consists of wingtip vortices, which refer to a pair of counter-rotating vortex pairs formed at the wingtips. These pairs are caused by pressure differences. The wake includes these vortex systems and all disturbed flow fields generated during their evolution. The vortex formation mechanism originates due to the pressure gradient between the low-pressure region caused by the accelerated airflow and high-pressure region over the upper and lower wing surfaces, respectively. This pressure difference not only provides lift but also generates the airflow from the lower surface to the upper surface around the wingtip, forming a vortex pair having equal intensity due to shear effects.

**Figure 1:**
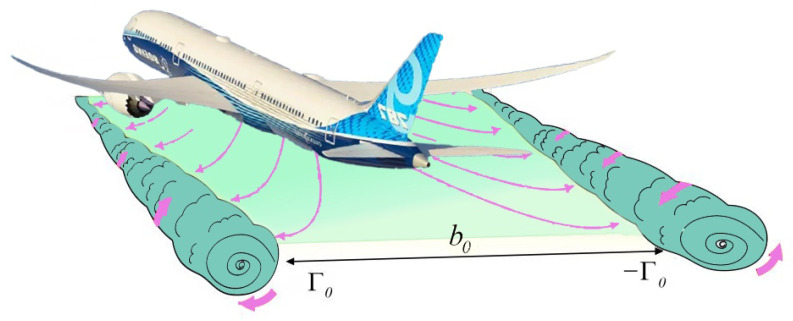
Schematic representation of wake vortex formation behind aircraft.

The dissipation characteristics of the vortex system can be divided into two primary stages: In the initial stage between 0 and 10 seconds, the vortex system exhibits a highly concentrated structure, with dense circulation in the vortex core region and stable structural features. Subsequently, between 10 and 20 seconds, the vortex system generates secondary vortex structures through Kelvin–Helmholtz instability under the influence of fluid viscosity [24]. These small-scale vortices promote the radial and axial diffusion and dissipation of the vortex system through friction and mixing in the shear layer. Figure 1 demonstrates the structure and evolution process of the wake vortex system. The symbol $b_{0}$ represents the initial separation distance between vortex cores, typically related to the aircraft’s wingspan. $\Gamma_{0}$ denotes the initial circulation of the left vortex (counterclockwise rotation), while $-\Gamma_{0}$ indicates the equal but opposite circulation (clockwise rotation) of the right vortex.

### 2.2. Principle of Doppler Lidar Detection

Measurements obtained based on the Doppler effect rely on the observation of frequency variations in light waves due to the relative motion between the source and the target. Doppler lidar, during its airspace scan, identifies backscattered laser signals originating from aerosol particles present in the aircraft’s wake. According to Equation (1), the Doppler frequency shift is related to the laser wavelength and the radial velocity of the particles as follows:

(1) $$\Delta f_{D}=\frac{2}{\lambda_{0}}V_{R}$$

where $\Delta f_{D}$ is the Doppler frequency shift that indicates the radial velocity of particles relative to the receiver. $\lambda_{0}$ is the wavelength of the laser. $V_{R}$ is the radial velocity of aerosol particles, which represents the forward or backward motion of particles relative to the receiver.

When an aircraft passes through the scanning region of a Doppler lidar system, the complex aerodynamic interaction between the aircraft and the surrounding air generates turbulent wake vortices near the wing tips. These vortices entrain aerosol particles, creating flow field characteristics that reflect the structure of the wake. By capturing the backscattered signals of Doppler frequency shifts from these particles, lidar achieves the measurement of radial wind velocity within the wake region.

In practical applications, the performance of Doppler lidar depends significantly on atmospheric conditions. For instance, in foggy weather, the scarcity of aerosol particles weakens the signal, while laser signals are significantly attenuated due to scattering and absorption by water droplets. Similarly, in rainy conditions, the quality of the backscattered signals further deteriorates. As a result, Doppler lidar performs best under clear weather conditions with high visibility.

Unlike systems that rely on visible light, Doppler lidar operates in the infrared band, enabling it to effectively collect data under both daytime and nighttime conditions. This feature makes it a versatile tool for wake vortex detection and monitoring, suitable for various operational scenarios.

**Figure 2:**
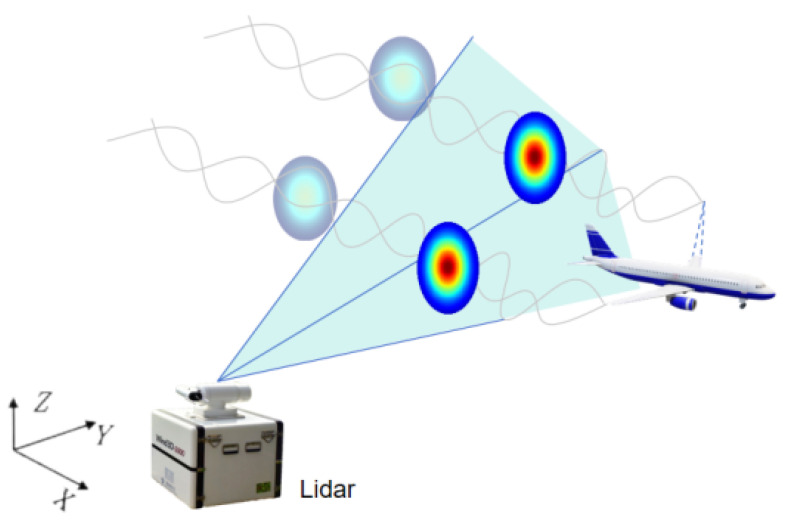
Schematic diagram of lidar scanning principle for wake detection.

Figure 2 illustrates the principle of Doppler lidar for aircraft wake detection. The figure demonstrates how laser beams interact with the turbulent flow field around the aircraft, providing critical information about the velocity distribution within the wake region.

For airport wake vortex observations, this study employs the Wind3D 6000 three-dimensional scanning Doppler LiDAR system. This system operates based on the principle of optical coherent pulsed Doppler frequency shift detection, which precisely measures wind velocities at various distances by analyzing the Doppler frequency shift of laser signals scattered back from atmospheric particles. Coherent pulsed Doppler technology offers advantages of high precision, long-range detection, and high spatial resolution, making it particularly suitable for wake vortex detection and tracking. Table 1 presents the key technical parameters of the Wind3D 6000 system. The 1.5 μm wavelength laser is eye-safe and provides a detection range of up to 6000 m, enabling long-duration tracking of wake vortices.

In Figure 2 illustrates how the radar utilizes laser beams to detect airflow disturbances induced by an aircraft’s wake vortex. The A320 serves as the example in this simulation, which assumes no background wind and adopts the Hallock–Burnham model [5]. The research focuses on the radial velocity distribution of the wake vortex. In this simulation, the A320’s specifications are as follows: a maximum takeoff weight (MTOW) of 40.5 tons (representing the heaviest weight at which the aircraft is certified for takeoff), a wingspan of 27.29 m, and a cruising speed of 69.96 m/s. The initial vorticity is calculated using the following formula:

(2) $$\Gamma_{0}=\frac{Mg}{\rho Vb_{0}}$$

In Equation (2), $\Gamma_{0}$ represents the initial circulation of the wake vortex, where M is the aircraft mass, g is the gravitational acceleration, ρ is the air density, V is the inflow velocity approximated as the aircraft’s flight speed, and $b_{0}$ denotes the initial separation distance between the wake vortex cores.

**Figure 3:**
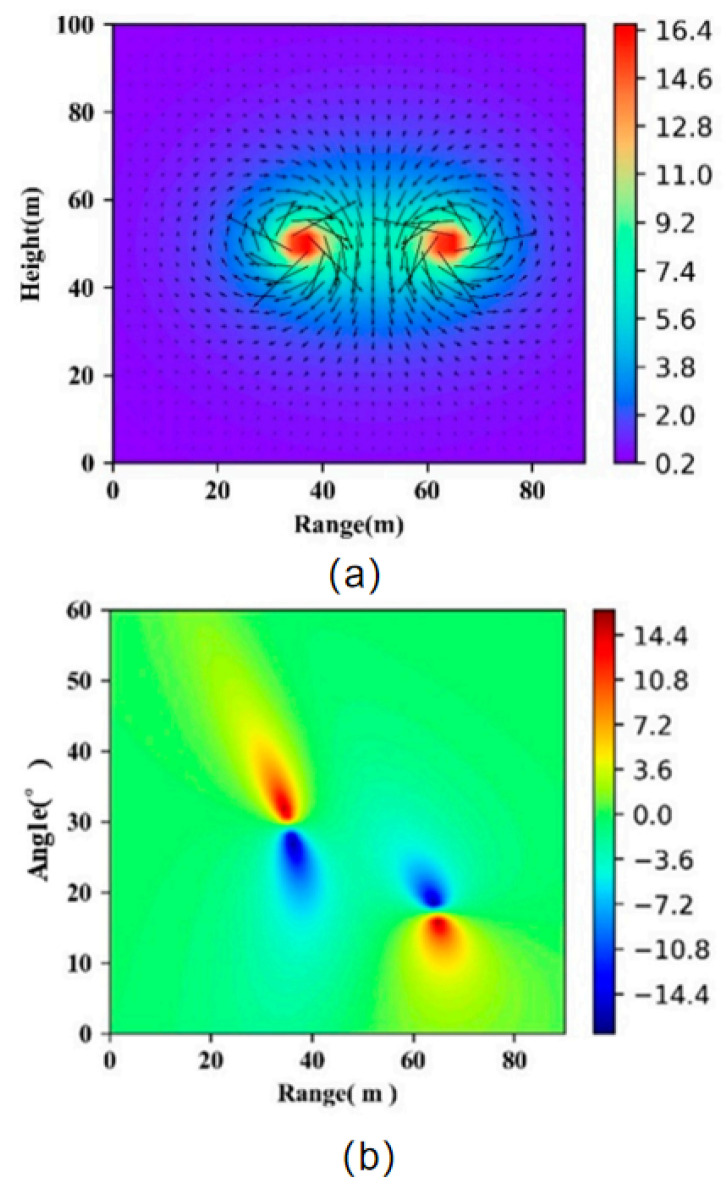
(**a**) Flow field and velocity distribution. (**b**) Jet cloud visualization.

The wake vortex generated by the A320 aircraft induces motion in the surrounding aerosol particles, which can be visualized in their velocity distribution and flow field. Figure 3 provides a detailed representation of these characteristics. Specifically, Figure 3a illustrates the flow field and velocity intensity of the wake vortex. The arrows indicate the flow direction, while the color bar represents the velocity magnitude. Regions in red correspond to higher velocity magnitudes, whereas regions in blue indicate lower velocities. The wake vortex exhibits a typical twin-vortex structure, with the maximum velocity intensity observed near the core of each vortex. Figure 3b presents the jet cloud image, where the horizontal axis denotes the horizontal distance between the LiDAR and the particles, and the vertical axis corresponds to the LiDAR scanning angle. The color bar represents the radial velocity, with red regions indicating positive radial velocities (particles moving away from the LiDAR) and blue regions indicating negative radial velocities (particles moving toward the LiDAR). When the LiDAR scans the origin of the wake vortex shown in Figure 3, an aerosol particle located at point P is considered, whose coordinates are denoted as (r,θ). Here, r defines the horizontal distance between the lidar and point P, while θ represents the elevation angle at point P. The radial velocity at this point can be determined using Equations (3) and (4):

(3) $${\vec{v}}_{t}={\vec{v}}_{t1}+{\vec{v}}_{t2}$$

(4) $$|{\vec{v}}_{r}|=\frac{\vec{OP}\cdot {\vec{v}}_{t}}{|\vec{OP}|}$$

### 2.3. Wake Vortex Field Detection

To obtain three-dimensional scanning data of a complex wind field and precise information on aircraft wake vortices, this experiment collected wind field data from a close-range site at the Shuangliu Airport, using the Wind3D 6000 lidar. The lidar has long-range detection capabilities, with a maximum detection range of over 6 km. Additionally, it is highly practical thanks to its compact size, lightweight design, and low energy consumption. Its commonly used scanning modes include the Range–Height–Indicator (RHI) mode and the Plan–Position–Indicator (PPI) mode. The former mode was selected for the cross-sectional scanning of wake vortices in this experiment.

**Figure 4:**
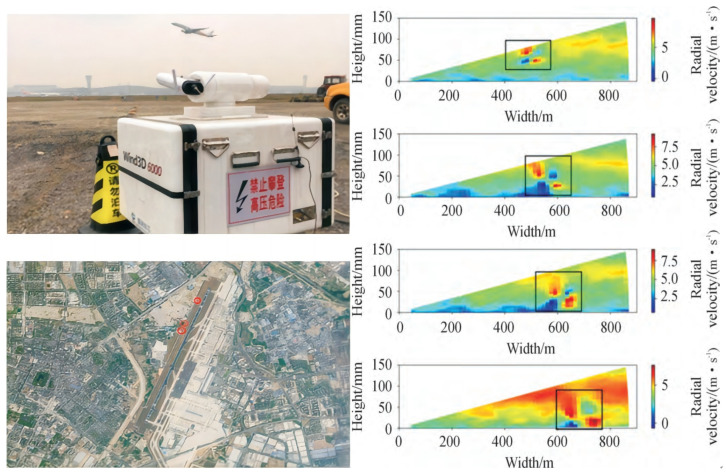
(**a**) Aircraft wake vortex observation site selection diagram. (**b**) A380 aircraft wake visualization.

Figure 4a shows the specific placement of the Doppler lidar during the actual detection process.

The intensity of wake vortices generated by different aircraft models and their evolution are closely related to the surrounding environment. To obtain ideal observation conditions, the lidar operating parameters must be configured based on factors such as airport terrain, weather conditions, and runway operations, as shown in Table 2. The lidar periodically scans the target detection area at a fixed scanning rate, which enables extensive collection of extensive wake vortex evolution data under various weather conditions and for different aircraft models.

The experimental campaign was conducted at Chengdu Shuangliu International Airport (CTU), southwest China (30°34′37″ N, 103°56′53″ E), from April to September 2023. The observation site was located 350 m from runway 02R/20L. Throughout the observation period, various atmospheric conditions were documented with temperatures ranging from 10.3 °C to 34.6 °C and relative humidity between 45% and 85%. Three atmospheric stability conditions were observed: neutral (61. 5%), weakly stable (23. 2%), and weakly unstable (15.3%). Wind conditions, critical for wake vortex behavior, included average speeds of 1.8 m/s to 7.3 m/s, with prevailing southwest and northeast directions. The dataset captures wake vortex evolution under light (<3 m/s, 38%), moderate (3–5 m/s, 42%), and strong (>5 m/s, 20%) crosswind scenarios, providing comprehensive coverage of wake characteristics across various types of aircraft operating at CTU.

Taking the A380 as an example, Figure 4b shows the wake vortex visualization results obtained on-site. The analysis of the wake vortex evolution reveals that, under the mutual induction of the vortices and the influence of environmental wind, the left and right vortices exhibit a gradually expanding trend in shape, while the strength of the vortex rings gradually weakens. Although the vortices retain a certain degree of overall counter-symmetry, this symmetric structure becomes increasingly unstable over time and eventually merges with the surrounding wind field.

## 3. Deep Learning Methods and Implementation

The network proposed in this paper offers significant advantages for rapid wake vortex identification using Doppler lidar data. While these data capture detailed velocity fields and fluid properties, they present challenges due to their complex multi-scale features, susceptibility to noise, and environmental interference. To address these limitations, we propose a hybrid architecture combining VGG16 and Inception modules.

VGG16 serves as an ideal backbone despite newer alternatives because its intuitive structure enhances interpretability for safety-critical aviation applications, and its moderate downsampling better preserves fine vortex structures. Our experiments confirm that VGG16 maintains competitive recognition accuracy compared to newer models such as ResNet and YOLOv5, while also achieving inference speeds suitable for real-time monitoring applications. This architecture leverages VGG16’s hierarchical feature extraction through stacked 3 × 3 convolutions to effectively capture velocity gradients and edge contours.

The integration of Inception modules complements VGG16 by employing a multi-branch structure with varied kernel sizes (1 × 1, 3 × 3). This design enables simultaneous extraction of both local details (small-scale vortices) and global characteristics (velocity distributions), while dimensionality reduction operations enhance computational efficiency. By combining these complementary strengths, our proposed model achieves superior identification efficiency and accuracy for wake vortex monitoring.

**Figure 5:**
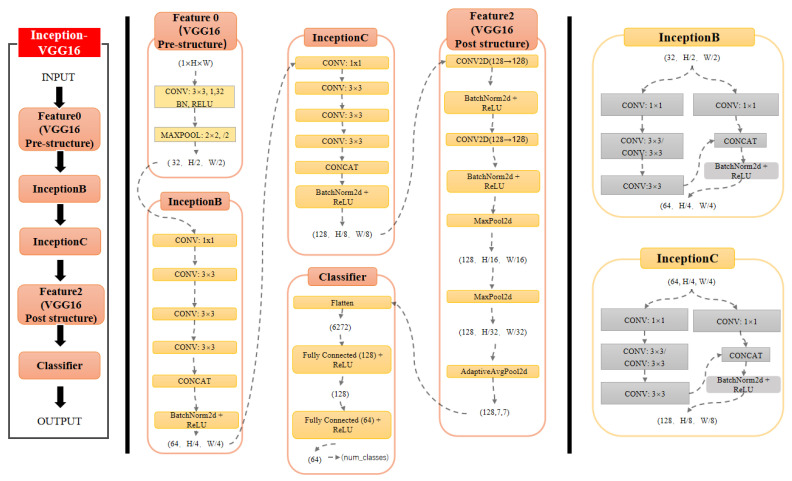
Inception-VGG16 network architecture diagram.

By combining the advantages of these two architectures, this paper proposes the Inception-VGG16 model: a wake vortex rapid identification network based on VGG16 and Inception modules. This model integrates improved Inception modules (named InceptionB and InceptionC, respectively) into the traditional VGG16 network to enhance the ability to extract multi-scale features while retaining the hierarchical feature extraction strengths of VGG16. The model is designed to efficiently process the complex, multi-scale, and multi-level features of wake vortices while improving robustness against noisy data. The structure of the network is illustrated in Figure 5.

### 3.1. Feature0 Module

To address the issues of insufficient feature extraction and inadequate adaptability to environmental changes in wake vortex identification tasks, this paper designs an efficient feature extraction module. The module consists of convolution, batch normalization, ReLU activation, and max pooling operations. Its primary function is to extract deep and multi-scale feature representations from wake vortex images, which enhance the ability to recognize wake vortex patterns while improving the model’s robustness and computational efficiency.

The mathematical structure of this module is given by the following expression:

(5) $$z=\mathrm{MaxPool}\left(\mathrm{ReLU}\left(\mathrm{BatchNorm}\left(\mathrm{Conv}(x;W,b)\right)\right)\right)$$

where the input feature map is denoted as *x*, while *W* and *b* represent the weights and biases of the convolutional layer, respectively. Futhermore, Conv denotes the convolution operation, BatchNorm represents the batch normalization operation, ReLU refers to the activation function, and MaxPool indicates the max pooling operation. Through step-by-step processing between layers, this module is capable of extracting critical edges, textures, and shape features from wake vortex images while eliminating redundant information.

**Figure 6:**
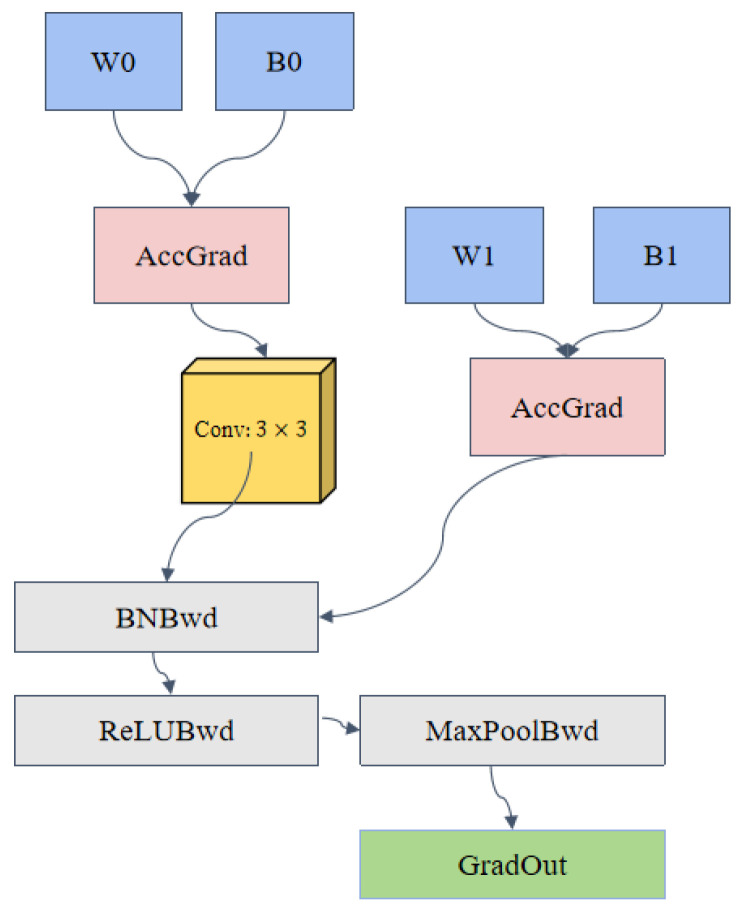
Network structure of the Feature0 module.

During the wake vortex identification process, this module uses convolution operations to extract local features of the wake vortex. The feature representation is further optimized using batch normalization and the activation function, while the max pooling operation compresses the feature map dimensions and retains the key information. This design effectively enhances the wake vortex feature extraction accuracy and improves the model’s training efficiency, providing reliable support for precise wake vortex pattern recognition. The network structure of the Feature0 Figure 6 shows the network structure of the Feature0 module.

### 3.2. Inception Module

In this network, two improved Inception modules, namely, the InceptionB and InceptionC modules, are designed to achieve parallel extraction of multi-scale features from wake vortex images:

1. **InceptionB Module**:
  This module consists of two parallel branches, whose functions are defined as follows:
  - **Branch 1**: Performs dimensionality reduction and feature extraction using a 1 × 1 convolution kernel.
  - **Branch 2**: Further extracts local features using a 3 × 3 convolution kernel and deepens the network’s non-linear representation capabilities through consecutive convolutional operations.
    The output feature channel number is 64.
2. **InceptionC Module**:
  This module builds upon the InceptionB module by expanding the number of output channels number to 128, further enhancing the diversity and expressive power of the features. It introduces a deeper convolutional structure to support the recognition of complex wake vortex patterns.

InceptionB and InceptionC modules adopt a branching design, combining convolution kernels with different receptive fields (e.g., 1 × 1 and 3 × 3) and fusing the multi-branch features through a concatenation operation. This design enables the network to capture both local details and global patterns of the wake vortex.

Considering the InceptionB module as an example, its mathematical structure can be described as follows, where the input feature map is denoted by x with dimensions (B, C, H, W):

Branch 1: 1 × 1 convolution branch:

(6) $$x_{1}={\mathrm{Conv}}_{1\times 1}\left(x;W_{1},b_{1}\right)$$

Here, $W_{1}$ and $b_{1}$ represent the weights and biases of the convolution kernel, respectively.

Branch 2: 1 × 1 convolution + three layers of 3 × 3 convolution:

(7) $$x_{2}={\mathrm{Conv}}_{3\times 3}\left({\mathrm{Conv}}_{3\times 3}\left({\mathrm{Conv}}_{3\times 3}\left({\mathrm{Conv}}_{1\times 1}\left(x;W_{2},b_{2}\right);W_{3},b_{3}\right);W_{4},b_{4}\right);W_{5},b_{5}\right)$$

Then, the outputs of Branch 1 and Branch 2 are concatenated along the channel dimension as follows:

(8) $$x_{\mathrm{out}}=\mathrm{Concat}\left(x_{1},x_{2}\right)$$

The concatenated features are processed through BatchNorm and ReLU activation as

(9) $$y=\mathrm{ReLU}\left(\mathrm{BatchNorm}(x_{\mathrm{out}})\right)$$

The mathematical structure of the InceptionC module is similar to that of InceptionB, but it features a higher number of output channels for each branch and introduces deeper convolutional layers.

**Figure 7:**
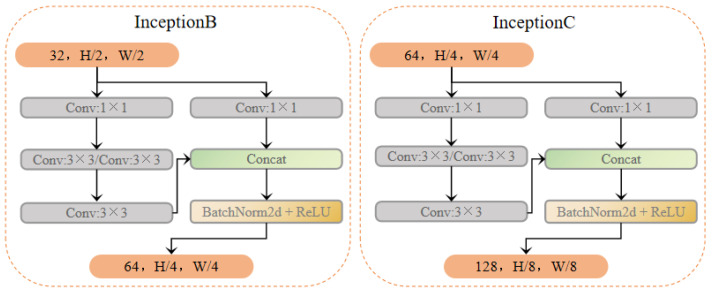
Network structure of the Inception module.

As the core component of the Inception-VGG16 model, the inception module effectively addresses the challenge of multi-scale feature extraction in wake flow recognition through its multi-branch parallel design and feature fusion mechanism. Additionally, its efficient computational design and powerful feature representation capabilities enable the model to excel in both accuracy and real-time performance for wake flow recognition. The improved InceptionB and InceptionC modules proposed in the paper provide innovative technical solutions for wake flow recognition tasks, as shown in Figure 7.

### 3.3. Classifier Module

The classification structure represents the backend of the improved Inception-VGG16 network proposed in this paper. Its primary function is to integrate and compress the large number of deep, multi-scale features extracted by the frontend feature extraction modules (including Feature0, InceptionB, and InceptionC) to ultimately achieve wake flow pattern classification and recognition. This module progressively maps high-dimensional features to low-dimensional space using fully connected layers and enhances the accuracy and robustness of wake flow recognition by combining the activation functions and regularization operations.

In wake flow recognition tasks, this structure flattens and compresses the high-dimensional features output by the frontend multi-scale feature extraction modules, retaining key feature information while removing redundant features. The fully connected layers progressively extract the characteristic patterns of wake vortices and map them to the wake flow category space, completing the classification of wake flow patterns. Dropout is applied as a regularization operation to improve the model’s robustness against complex backgrounds and noisy data. Finally, the Softmax activation function outputs the probability distribution of wake flow patterns, enabling fast classification of wake flows.

**Figure 8:**
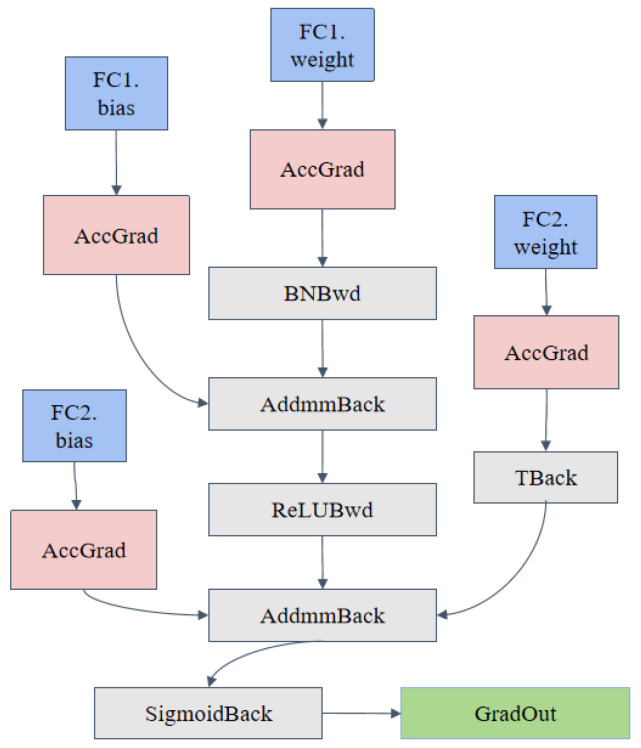
Network structure of the Classifier module.

The comprehensive formula for the entire classification structure can be expressed comprehensively via the following expression:

(10) $$y=\mathrm{Softmax}\left(W_{3}\cdot \mathrm{ReLU}\left(W_{2}\cdot \mathrm{Dropout}\left(\mathrm{ReLU}\left(W_{1}\cdot x_{\mathrm{flat}}+b_{1}\right)\right)+b_{2}\right)+b_{3}\right)$$

where y represents the predicted category distribution of wake vortices, and the Softmax function is responsible for converting the output into a probabilistic form. The input $x_{\mathrm{flat}}$ has dimensions of (B,C,H,W), where $W_{1}$ and $b_{1}$ represent the weight matrix and bias term of the first fully connected layer, respectively, and ReLU is the activation function. Figure 8 shows the structure of the classification network.

In summary, the Inception-VGG16 hybrid deep learning network proposed in this paper can efficiently process and accurately classify wake flow data by combining the multi-scale feature extraction capability of the Inception modules with the deep feature representation ability of VGG16. The collaborative operations of the Feature0 module, the improved InceptionB and InceptionC modules, and the hierarchical classification structure enable the model extract key wake flow features in complex backgrounds and noisy environments, resulting in high classification accuracy and robustness. Moreover, the model’s high inference efficiency meets the real-time requirements of practical aviation wake flow monitoring tasks.

### 3.4. Model Training and Optimization

Due to the significant dynamic variations in wind speed within the background wind fields of wake flows, this study applied standardization to the collected data to eliminate the influence of dimensions on the network input data. The standardized data not only accelerate the gradient descent process during the training of the convolutional neural network (CNN) but also effectively improve the convergence efficiency of the model. After preprocessing, the dataset was randomly divided into a training set, validation set, and test set, with distribution ratios of 60%, 20%, and 20%, respectively. The training set was used for learning the model weights, the validation set was applied during training for hyperparameter tuning, and the test set was used for model evaluation to obtain the final experimental results.

The Adam optimizer, known for its adaptive gradient descent approach, was chosen for model training due to its simplicity, computational efficiency, and low memory consumption. It is commonly used in domains like computer vision and NLP, and in this study, it was configured with a learning rate of 0.001, a momentum of 0.9, and a weight decay of $1\times 10^{-4}$. Cross-entropy loss is utilized to evaluate the difference between the predictions and true probabilities, with smaller values indicating better model performance. The expression for the cross-entropy loss is as follows:

(11) $$H\left(p,q\right)=-\sum_{x}p\left(x\right)\log q\left(x\right)$$

By combining the above optimization algorithm and the loss function, the model can learn data features more efficiently and improve the prediction performance.

## 4. Experiments and Results

Experiments were performed on a Windows 11 system with Python and PyTorch 2.4.0 as the implementation frameworks. The hardware setup included an NVIDIA GeForce RTX 4090 GPU, a 13th Gen Intel® Core™ i7-13700K processor, and 32GB of RAM.

The dataset for this study was constructed based on wake flow observation experiments conducted by the team at Chengdu Shuangliu International Airport (CTU). The experiments utilized the Wind3D 6000 Doppler LiDAR, with equipment deployed at various observation points along the runway, and a total of 3530 wind field samples collected to form the dataset. Among these samples, those containing aircraft wake vortices are labeled as positive samples (T), while those without wake vortices are labeled as negative samples (F). It is important to note that, to avoid unnecessary warnings, wake vortices that no longer pose a threat to subsequent aircraft during the dissipation phase are classified as negative samples.

**Figure 9:**
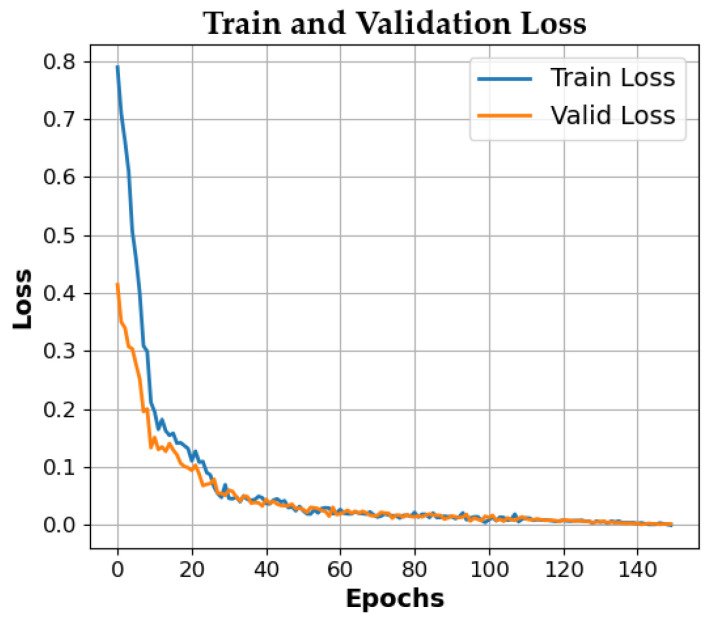
Training and validation loss values versus iterations.

The preprocessed training set data were fed into the Inception-VGG16 model for training. Figure 9 shows the iterative changes in the training and validation loss values versus epochs during the training process.

The effectiveness of the constructed target recognition network is further validated by comparing its performance with common target recognition networks, such as VGG16, SVM, KNN, and RF. The following metrics are used for the performance comparison:

Classification Accuracy (Accuracy):

(12) $$Accuracy=\frac{TP+TN}{TP+TN+FP+FN}$$

Precision:

(13) $$Precision=\frac{TP}{TP+FP}$$

Recall:

(14) $$Recall=\frac{TP}{TP+FN}$$

F1-score, which considers the balance between precision and recall:

(15) $$F1=2\cdot \frac{Precision\cdot Recall}{Precision+Recall}$$

In the above expressions, TP refers to the correctly identified positive samples, while TN denotes the correctly classified negative samples. Furthermore, FP indicates negative samples that are misclassified as positive (Type I error), and FN represents positive samples that are misclassified as negative (Type II error).

The experimental results are shown in Table 3. Achieving the highest accuracy (0.988), precision (0.972), and F1-score (0.966), the Inception-VGG16 model excelled in all evaluation metrics. This highlights the effectiveness of the developed Inception-VGG16 network in identifying aircraft wake vortices and reducing the occurrence of false alarms.

**Figure 10:**
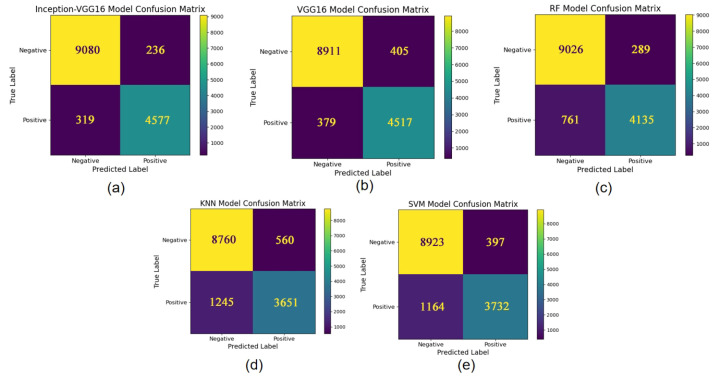
Confusion matrices. (**a**) Inception-VGG16 model confusion matrix; (**b**) VGG16 model confusion matrix, (**c**) RF model confusion matrix; (**d**) KNN model confusion matrix; (**e**) the SVM model confusion matrix.

The binary classification performance of each model is further analyzed using confusion matrices, providing insights into their capabilities across different categories. As shown in Figure 10 shows, deep learning models like VGG16 and Inception-VGG16 exhibited outstanding results, especially under conditions of imbalanced positive and negative sample distributions. Note that the dataset used in this study, derived from Shuangliu Airport, represents a typical real-world scenario with considerable variability. Traditional machine learning models (e.g., SVM, KNN, and RF) performed adequately but struggled to balance precision and recall effectively.

To further validate the effectiveness of our proposed method, we compared the Inception-VGG16 model proposed in this paper with the related work mentioned in the Introduction section. Compared to traditional machine learning methods (SVM, KNN, RF) and single deep networks (VGG16), our model achieved the best performance in metrics such as accuracy, precision, recall, and F1-score. In particular, compared to our previously proposed improved GoogLeNet-based model [23] (accuracy of 98.44%), the Inception-VGG16 architecture in this article (accuracy of 98. 8%) still shows performance improvement: (1) The hybrid architecture combining VGG16’s hierarchical feature extraction with Inception modules’ multi-scale capabilities addresses the multi-scale nature of wake vortex features more effectively than single-architecture approaches. (2) The improved InceptionB and InceptionC modules in our model can extract multi-scale features in parallel, enhancing the ability to recognize wake vortices in complex backgrounds. (3) VGG16’s hierarchical structure performs deep feature extraction through multiple layers of convolution and pooling operations, further improving the model’s ability to express wake vortex features.

In addition to surpassing traditional machine learning and single deep learning models, our Inception-VGG16 network demonstrates robust and reliable performance on real-world wake vortex datasets. Specifically, on the Chengdu Shuangliu Airport dataset, our model achieves a recognition accuracy of 98.8%, with a precision, recall, and F1-score of 97.2%, 96.1%, and 96.6%, respectively. These results reflect the model’s strong capability to accurately detect and classify wake vortices under complex and variable conditions.

For further comparison, recent studies such as Shen et al.’s study in [18] reported a YOLOv5s-based approach for wake vortex recognition that achieved an accuracy of 92.4% and an inference speed of 416.7 FPS. While such methods exhibit impressive real-time performance, their accuracy on similar tasks remains significantly lower than that of our Inception-VGG16 model. Therefore, our proposed approach not only delivers higher recognition accuracy but also maintains efficient inference, making it highly suitable for practical aviation safety monitoring.

Based on the confusion matrices of the five models, it is evident that the Inception-VGG16 model demonstrates the best performance. It has the lowest number of misclassifications and achieves high accuracy in both positive and negative sample classification, making it well suited for tasks with strict classification requirements. The VGG16 model exhibits a balanced overall performance, with strong classification capabilities for both positive and negative samples. Although its error rate is slightly higher than that of Inception-VGG16, it remains an excellent choice. The Random Forest (RF) model performs exceptionally well in negative sample classification but shows slightly lower recall for positive samples, which may pose a risk of missed detections in certain tasks. In contrast, the performance of the KNN and SVM models is somewhat inferior, particularly in terms of recall for positive samples, where significant improvement is needed to reduce the likelihood of missed detections.

To gain deeper insights into the model’s limitations and guide future improvements, we conducted a detailed analysis of misclassification cases produced by the Inception-VGG16 network, focusing on typical false positive and false negative scenarios.

In false positive cases, we identified two main patterns of misclassification. The first pattern occurred when natural turbulence formations at cloud layer edges exhibited structural features similar to wake vortices, causing the model to incorrectly identify them as wake vortices. These natural turbulence patterns often presented rotational patterns and linear structures that resembled genuine wake vortex signatures. The second common false positive scenario involved high-contrast turbulent regions near aircraft. In these cases, local air disturbances caused by the aircraft shared visual characteristics with actual wake vortices, leading to model confusion. These examples demonstrate that the model still faces challenges when distinguishing wake vortices from natural turbulence phenomena that present similar texture and shape features.

In our false negative analysis, we also identified two primary scenarios. First, when wake vortices were in their dissipation phase, their structural features became less pronounced with significantly reduced intensity, making it difficult for the model to recognize these weak signal features. This was particularly common after 20 s of vortex formation, when the vortices had begun to break down into smaller turbulent structures. Second, low-contrast wake vortices in complex cloud backgrounds were often obscured by environmental noise. In these situations, the wake vortex feature information blended with the background, making feature extraction exceptionally challenging and preventing the model from successfully identifying existing wake vortices.

Based on the analysis of these error cases, we propose the following improvement strategies: (1) enhancing the training dataset diversity, particularly by increasing samples with weak wake signals and specific background interference conditions; (2) introducing attention mechanisms to improve the model’s ability to differentiate between wake vortices and natural turbulence structures; (3) improving feature fusion strategies to increase the model’s sensitivity to low-contrast scenarios; and (4) considering the introduction of temporal information processing mechanisms to better capture wake vortex evolution characteristics. These targeted improvements are expected to further enhance the model’s robustness in real-world application environments.

Through systematic analysis of these error cases, we have gained a deeper understanding of the current limitations of the Inception-VGG16 model when processing complex environments and boundary cases, providing clear guidance for future research directions. These insights are significant for improving the reliability of the model in practical aviation safety monitoring applications.

Our experimental results demonstrate that the proposed Inception-VGG16 architecture effectively addresses the three key challenges identified in the introduction. First, regarding the limited capability of single network architectures to handle multi-scale wake vortex features, our hybrid network successfully integrates the parallel multi-scale processing strength of Inception modules with VGG16’s hierarchical feature representation, achieving an impressive 98.8% accuracy in wake vortex recognition. Second, concerning the need for improved feature extraction and noise suppression under complex backgrounds, our model demonstrates superior performance with 97.2% precision and 96.1% recall, indicating robust feature representation even in challenging conditions with minimal false positives and false negatives. Third, our architecture successfully balances real-time performance and accuracy within a single model, maintaining state-of-the-art recognition performance while achieving inference speeds suitable for real-time monitoring applications. These results validate the effectiveness of our hybrid approach in overcoming the limitations of existing wake vortex recognition methods.

To further clarify the physical interpretability of the proposed Inception-VGG16 model, we conducted an in-depth analysis of the feature responses at different network layers during the wake vortex recognition process. By tracing the activation patterns from the initial convolutional layers through to the deeper modules, we observed that the model consistently emphasizes image regions where the Doppler lidar data present abrupt changes in radial velocity, as well as contiguous structures that resemble the classic vortex pair configuration.

In practical terms, this means the model is sensitive to the velocity discontinuities and rotational patterns that are characteristic of wake vortices according to aerodynamic theory. For example, in several typical cases, we found that the network’s intermediate representations tended to highlight the spatial zones corresponding to the expected positions of vortex cores, and were less influenced by homogeneous background flow.

This layer-wise analysis demonstrates that the model’s decision-making process is aligned with the established physical understanding of wake vortex phenomena. By focusing on these physically meaningful signatures, the network’s predictions are not only accurate but also interpretable within the context of fluid mechanics, which is important for building user trust in operational settings.

In addition to controlled experiments, we evaluated the practical effect of the proposed method in the context of real airport operations. The trained Inception-VGG16 model was directly applied to a series of new Doppler lidar observations that reflected the variability of actual airport environments, including changes in weather, wind direction, and aircraft types.

During this process, the model was able to adapt to different operational scenarios without the need for additional calibration or manual intervention. For instance, when exposed to lidar data collected during periods of rapidly shifting wind or mixed aircraft traffic, the model maintained a consistent ability to identify wake vortex events and provide timely outputs.

Moreover, discussions with airport technical staff revealed that the model’s output could be readily interpreted and integrated with existing safety monitoring workflows. In practice, the method helped operators quickly distinguish between genuine wake vortices and other transient disturbances, enhancing situational awareness. Although certain edge cases, such as overlapping turbulence from multiple sources, still pose challenges, the overall experience demonstrates the method’s suitability for operational deployment and its potential to improve the efficiency and reliability of wake vortex monitoring in real-world environments.

## 5. Conclusions

In this paper, a novel hybrid deep learning architecture, the Inception-VGG16, was proposed to address the challenges of aircraft wake vortex recognition. The model effectively combined the multi-scale feature extraction capabilities of the Inception modules with the hierarchical feature representation of the VGG16 network. The network achieved robust parallel extraction of multi-scale features by incorporating the improved InceptionB and InceptionC modules, while the VGG16 structure ensured deep feature representation. The proposed architecture demonstrated excellent performance in recognizing wake vortex patterns, especially in complex environments with noise interference. The experimental results validated the effectiveness of the Inception-VGG16 model. Compared with traditional machine learning models, e.g., SVM, KNN, and RF, and single deep learning models, e.g., VGG16, the proposed model achieved the highest accuracy, precision, recall, and F1-score values of 98.8%, 97.2%, 96.1%, and 96.6%, respectively. These improvements highlighted the model’s superior ability in both feature extraction ability and noise robustness.

Furthermore, the model’s inference efficiency satisfies the real-time requirements of wake vortex monitoring tasks, making it an ideal candidate for aviation safety applications. In conclusion, the Inception-VGG16 network provides an innovative and effective approach to address the challenges of wake vortex recognition. It offers a promising technical direction for improving aviation safety systems and performing further research into deep learning applications in the field of aeronautics.

Despite its outstanding performance, the proposed architecture has a few limitations. The dataset used in this research was collected at the Shuangliu Airport. Although it represents a realistic and dynamic environment with considerable variability, it does not fully consider all environmental conditions that might occur in real-world scenarios. In future work, we plan to expand the dataset to include a broader range of atmospheric conditions and diverse aircraft types. Additionally, further optimization of the model’s computational efficiency will be further improved to enhance its applicability in resource-constrained environments, such as onboard aircraft systems or remote sensing platforms.

In conclusion, the Inception-VGG16 network provides an innovative and effective approach to addressing the challenges of wake vortex recognition. It offers a promising technical pathway for advancing aviation safety systems and paves the way for further research into deep learning applications in the field of aeronautics.

## Figures and Tables

**Table 1:** Wind3D 6000 LiDAR specifications.

Metric Items	Parameter Value
Laser Wavelength	1.5 μm, invisible and safe for human eyes
Radial Detection Range	45 m–6000 m
Radial Distance Resolution	15 m/30 m/user-defined
Data Refresh Rate	1 HZ–10 HZ
Radial Wind Speed Measurement Range	–37.5 m/s–37.5 m/s
Wind Speed Measurement Accuracy	≤0.1 m/s
Scan Servo Accuracy	0.1°
Scan Modes	fixed point/DBS/VAD/RHI/PPI/CAPPI,
	script programming
Weight	<90 kg

**Table 2:** Radar operating parameters (RHI mode).

Parameters (Unit)	Value
Azimuth angle (°)	112
Scanning rate (°/s)	1
Elevation range (°)	0–10
Elevation angle resolution (°)	0.2 ± 0.03
Detection radial range (m)	45–885
Longitudinal resolution (m)	15
Distance between points A and B (m)	503
Distance between points B and C (m)	1468

**Table 3:** Comparison of object recognition networks.

Model	Accuracy	Precision	Recall	F1-Score
KNN	0.909	0.640	0.714	0.930
SVM	0.917	0.763	0.724	0.743
VGG16	0.984	0.951	0.959	0.955
RF	0.923	0.778	0.752	0.765
Inception-VGG16	0.988	0.972	0.961	0.966

## Data Availability

The authors confirm that the data supporting the findings of this study are available within the article.
